# The association of left ventricular histologically verified myocardial fibrosis with pulmonary hypertension in severe aortic stenosis

**DOI:** 10.1177/02676591211042733

**Published:** 2021-09-15

**Authors:** Birute Gumauskiene, Egle Drebickaite, Dalia Pangonyte, Jolanta Justina Vaskelyte, Lina Padervinskiene, Povilas Jakuska, Algimantas Budrikis, Rokas Ereminas, Egle Ereminiene

**Affiliations:** 1 Department of Cardiology, Medical Academy, Lithuanian University of Health Sciences, Kaunas, Lithuania; 2 Institute of Cardiology, Medical Academy, Lithuanian University of Health Sciences, Kaunas, Lithuania; 3 Department of Radiology, Medical Academy, Lithuanian University of Health Sciences, Kaunas, Lithuania; 4 Department of Cardiac, Thoracic and Vascular Surgery, Medical Academy, Lithuanian University of Health Sciences, Kaunas, Lithuania

**Keywords:** aortic stenosis, pulmonary hypertension, myocardial fibrosis

## Abstract

**Objectives:**

To evaluate the association between histologically verified left ventricular (LV) myocardial fibrosis (MF) and its bio- and functional markers with pulmonary hypertension (PH) in severe aortic stenosis (AS).

**Methods:**

About 34 patients with isolated severe AS underwent 2D echocardiography, cardiac magnetic resonance (CMR) imaging, and plasma NT-proBNP evaluation before aortic valve replacement (AVR). LV measurements were analyzed by CMR and LV strain using feature tracking software (Medis Suite QStrain 2.0). Myocardial biopsy sampled at the time of AVR was assessed by a histomorphometric analysis. PH was defined as pulmonary artery systolic pressure (PASP) ⩾ 45 mm Hg.

**Results:**

Patients with severe AS and PH (mean PASP 53 ± 3.7 mm Hg) had higher extent of diffuse MF versus patients without PH (12 (10.4–12.7)% vs 6.6 (4.6–8.2)% (p = 0.00)). The extent of diffuse MF correlated with LV dilatation (*r* = 0.7, p = 0.02), indices of LV dysfunction (lower ejection fraction (*r* = −0.6, p < 0.001), global longitudinal (*r* = −0.5, p = 0.02) and circumferential strain (*r* = −0.5, p = 0.05), elevated NT-proBNP (*r* = 0.5, p = 0.005) and elevated PASP (*r* = 0.6, p < 0.001)). Histological MF > 10% (AUC 94.9%), LV global longitudinal strain > −15.5% (AUC 86.3%), and NT-proBNP > 2090 ng/l (AUC 85.1%) were independent predictors of PH in severe AS.

**Conclusions:**

The extent of diffuse myocardial fibrosis in combination with reduced longitudinal left ventricular strain and increased plasma levels of NT-proBNP relates to pulmonary hypertension in severe aortic stenosis.

## Background

The presence of pulmonary hypertension (PH) in patients with severe aortic stenosis (AS) is quite common and associated with poor prognosis, therefore the identification of its determinants is clinically relevant.^1,2^ The progression of AS grade leads to left ventricular (LV) hypertrophy, and the development of LV myocardial fibrosis (MF). These changes are supposed to affect the severity of LV remodeling and dysfunction.^3–6^ However, the data of the MF impact in the development of PH in patients with severe AS is lacking.

Although myocardial biopsy is the golden standard for detecting MF, it is not routinely recommended in clinical practice. With the improvement of advanced imaging techniques together with the evaluation of plasma biomarker levels, it became possible to assess MF noninvasively. CMR late gadolinium enhancement techniques permit the revelation of advanced myocardial alterations—replacement fibrosis. However, the detection of early and possibly reversible diffuse MF, involving the subendocardial layers, is more important in establishing prognosis and treatment strategies. CMR feature tracking or speckle tracking echocardiography derived LV global longitudinal strain is a functional marker that demonstrates significant correlation with histological burden of MF and is considered as a marker that allows detecting subclinical damage.^7–10^ Plasma biomarkers (natriuretic peptides) relation with MF is also shown in many different cardiac pathologies.^11–14^ However, the data about the association between the extent of the histologically proven diffused myocardial fibrosis, LV function and mechanics, blood biomarkers, and PH in patients with valvular disease is scarce.

Therefore, the purpose of this study was to evaluate the association of histologically verified left ventricular MF and its bio- and functional markers with PH in severe AS.

## Methods

### Study population

The study included 34 patients with symptomatic severe AS, defined as an aortic valve area less than or equal to 1 cm^2^. Before participation in the study, all patients gave their informed consent. The study was approved by Kaunas Regional Biomedical Research Ethics Committee, No. BE–2–8, issued on October 29th, 2014. According to the presence or absence of PH (pulmonary artery systolic pressure (PASP) cut-off value of 45 mm Hg), patients were divided into two groups. Patients with documented coronary artery disease, chronic obstructive pulmonary disease, atrial fibrillation, or moderate to severe mitral regurgitation were excluded from the study.

### Transthoracic echocardiography

2D echocardiography was performed by an expert single investigator with a Vivid 7 ultrasound scanner (GE Vingmed Ultrasound AS N-3190, Horten, Norway), according to current echocardiography recommendations.^15^ The aortic valve area (AVA) was calculated using the continuity equation using the flow velocities in the LV outflow tract and across the valve. PH was defined as an estimated PASP ⩾ 45 mm Hg based on tricuspid valve regurgitation flow velocity ⩾3.0 m/s and the presence of other echocardiographic signs of PH.^16^

### Cardiac magnetic resonance imaging measurements

Cardiac magnetic resonance imaging (CMR) was performed using a 1.5T whole-body system (Siemens Aera, Siemens Medical Solutions; Erlangen, Germany) blinded to clinical parameters. LV volume and mass analysis were performed in standard short-axis cine images using CMR analysis software system (syngo.via; Siemens Healthcare). LV fibrosis expansion was assessed using late gadolinium enhancement (LGE) sequence after administrating the infusion of gadolinium (0.2 mmol/kg). To assess LGE quantitatively, short-axis slices were inspected visually, the area of LGE was traced manually, and fibrosis area results were expressed as a percentage of myocardial mass.

### Feature tracking analysis

CMR images were analyzed using commercial feature tracking (FT) software package (Medis Suite QStrain 2.0; Medis Medical Imaging Systems bv). Endocardial contours of LV in end-diastole and end-systole were marked semi-automatically (or corrected manually if necessary) throughout the cardiac cycle on standard CMR balanced steady-state free precession (bSSFP) sequences. LV global longitudinal strain (GLS) was calculated by averaging the strain curves of long axis views and LV global circumferential strain (GCS) from the short-axis views.

### NT-proBNP

NT-proBNP blood samples were obtained from all patients 24 hour before the aortic valve replacement (AVR). The samples were centrifuged for 1 hour and the plasma serums were stored at −80°C before being tested using the chemiluminescent immunoassay analyzer “PATHFAST” (*Mitsubishi Chemical Medience Corporation*, Tokyo, Japan).

### Endomyocardial biopsy

In patients undergoing AVR, 34 biopsy samples were obtained from the basal LV septum. Samples were immediately fixed in 10% buffered formalin. Paraffin-embedded sections were stained with Pickro-Mallory method. Cross-sectional images were analyzed using Pannoramic Viewer Computer System software. After elimination of the artifacts and perivascular fibrosis, area occupied by interstitial fibrosis was marked in black and then the area of interstitial collagen was expressed a percentage of total endomyocardial area. Two blinded and independent observers analyzed all the specimens. For the standardization of the study, the measurements were repeated three times, with a less than 5% error.

### Statistical analysis

All statistical analyses were performed using SPSS 23.0 (software (*SPSS Inc, Chicago*, IL, USA)). Continuous variables were expressed as median (interquartile range) and categorical variables were described as a percentage (number). The Mann-Whitney test was used to compare quantitative sizes of two independent samples. Chi-square (Fisher’s Exact Test) test was used to compare categorical variables. Bivariate correlations between study variables were calculated as needed by Pearson or Spearman’s rank correlation. Results were considered statistically significant when the two-tailed p-value was <0.05. The incremental value of all parameters in predicting the development of SPAP ⩾ 45 mm Hg was assessed in terms of the construction of receiver-operating characteristic (ROC) curves.

## Results

The study included 34 patients (55.9% male, mean age 70 (64–75) years) with symptomatic severe AS. According to the presence or absence of PH, with PASP cut*-*off value of 45 mm Hg derived by 2D echocardiography, nine patients (26.5%) had severe AS in combination with PH (mean PASP 53 ± 3.7 mm Hg).

Baseline demographic and clinical characteristics of the study cohort are summarized in Table 1.

**Table 1.** Demographic, clinical characteristics, left ventricular geometry, and function parameters and levels of NT-proBNP of the study patients.

	Overall (*n* = 34)	PASP < 45 mm Hg (*n* = 25)	PASP ⩾45 mm Hg (*n* = 9)	p Value
Age, years	70 (64–75)	69 (63–75)	75 (67–77)	0.17
Sex, male *n* (%)	19 (55.9)	12 (63.2)	7 (36.8)	0.12
Arterial hypertension, *n* (%)	28 (82.4)	20 (71.4)	8 (28.6)	0.48
Diabetes mellitus, *n* (%)	2 (5.9)	1 (50)	1 (50)	0.46
Body mass index, kg/m^2^	29 (25–32)	30 (25–32)	27 (23–34)	0.54
Glomerulal filtration rate, ml/min/1.73^2^	83 (68–101)	69 (91–103)	77 (61–83)	0.10
Aortic valve area, cm^2^	0.81 (0.7–1.0)	0.84 (0.68–1.1)	0.79 (0.64–1.0)	0.83
LV EDVi, ml/m²	125 (78–147.6)	90 (82.5–103)	140 (120–160)	<0.001
LV ESVi, ml/m²	47 (26–61)	32 (27–36)	55 (55–60)	<0.001
LV EF, %	56 (42–63)	61 (47–68)	46 (42–54)	0.03
LV MMi, g/m^2^	112 (84–146)	103 (82–130)	131 (118–148)	0.005
LV GLS, %	−15.5 (−22.5– (−14.8))	−21.1 (−23.4– (−17.8))	−14.0 (−14.9– (−8.9))	0.004
LV GCS, %	−26 (−18– (−31.2))	−32.0 (−36.1– (−20.8))	−16.4 (−15.4– (−10.9))	0.004
LGE midwall fibrosis, %	3.3 (1.9–7.8)	1.3 (1.2–1.48)	7.8 (5.6–8.0)	0.005
NT-proBNP, ng/l	1947 (595–5956)	522 (322–917)	2646 (936–7748)	0.02

PASP: pulmonary artery systolic pressure; LV: left ventricular; EDVi: end-diastolic volume index; ESVi: end-systolic volume index; EF: ejection fraction; MMi: myocardial mass index; GLS: global longitudinal strain; GCS: global circumferential strain; LGE: late gadolinium enhancement; NT-proBNP: N-terminal pro-hormone of brain natriuretic peptide

Patients in both groups were similar in terms of age, sex, body mass index, and with the same number of comorbidities (arterial hypertension, diabetes, and renal insufficiency) (p > 0.05). Patients with elevated PASP had larger LV volumes (LV EDVi (p = 0.015) and LV ESVi (p = 0.013)), increased LV MMi (p = 0.05), impaired LV systolic function (lower EF (p = 0.03), lower LV LGS (p = 0.014), and LV GCS (p = 0.016)) when compared with patients without PH.

Moreover, the extent of left ventricular LGE midwall fibrosis area was larger in patients with PH (7.8 (5.6–8.0)% vs 1.3 (1.2–1.48)%, p = 0.005).

**Figure 1.**
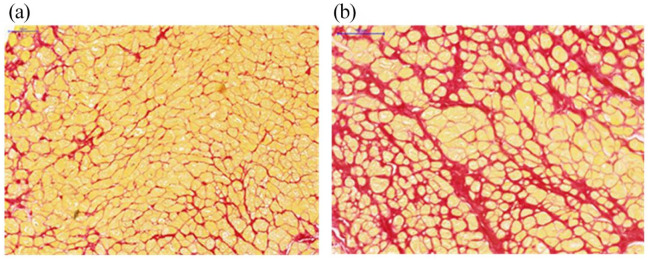
Histology from endomyocardial biopsies: (a) mild diffuse myocardial fibrosis expression in patient with aortic stenosis without pulmonary hypertension and (b) severe myocardial fibrosis expression in patient with severe aortic stenosis and pulmonary hypertension.

Histomorphometric analysis revealed that patients with severe AS and PH had higher extent of diffuse MF versus patients without PH (12 (10.4–12.7)% vs 6.6 (4.6–8.2)% (p < 0.001)) (Figure 1).

Increased plasma levels of NT-proBNP were observed in patients with PH compared to patients without PH (p = 0.02) (Table 1). NT-proBNP correlated with the extent of diffuse left ventricular MF (*r* = 0.5, p = 0.005) and elevated pulmonary artery systolic pressure (*r* = 0.6, p < 0.001).

**Figure 2.**
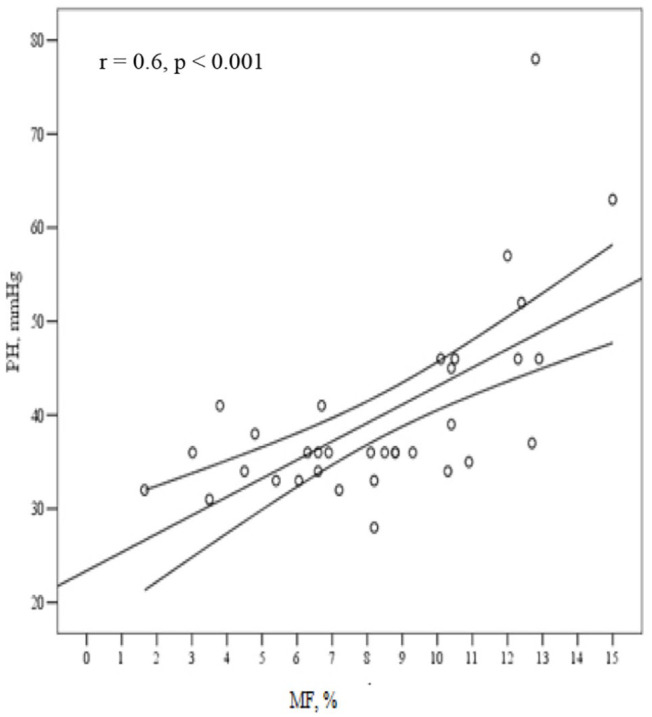
Association of histological left ventricular myocardial fibrosis with elevated pulmonary artery systolic pressure (PASP ⩾ 45 mm Hg). PH: pulmonary hypertension; MF: extent of left ventricular diffuse myocardial fibrosis;

The extent of diffuse MF demonstrated the association with CMR derived indices of LV volumes, ejection fraction, LV deformation parameters (Table 2) as well as with elevated PASP (*r* = 0.6, p < 0.001) (Figure 2). However, no correlation was found between histologically verified and LGE established replacement fibrosis (p = 0.08).

**Table 2.** Correlation of left ventricular diffuse myocardial fibrosis extent with indices of left ventricular geometry and function.

CMR parameters	*r*	p Value
LV EDVi, ml/m²	0.7	0.02
LV MMi, g/m²	0.7	0.02
LV EF, %	−0.6	<0.001
LV GLS, %	−0.5	0.02
LV GCS, %	−0.5	0.05
LGE fibrosis, %	0.2	0.08

LV: left ventricular; EDVi: end-diastolic volume index; MMi: myocardial mass index; EF: ejection fraction; GLS: global longitudinal strain; GCS: global circumferential strain.

ROC analysis date was constructed to evaluate the diagnostic accuracy of diffuse MF extent, indices of LV function and levels of NT-proBNP to predict the presence of PH in patients with severe AS. By regression analysis, histological MF > 10% (AUC 94.9%, sensitivity 100%, specificity 84%), LV GLS > −15.5% (AUC 85.1%, sensitivity 85.7%, specificity 88.9%), and NT-proBNP > 2090 ng/ml (AUC 85.1%, sensitivity 85.7%, specificity 88.9%) were found to be strong independent predictors of elevated pulmonary artery systolic pressure (Table 3).

**Table 3.** Receiver-operating characteristic (ROC) curve analysis of diffuse myocardial fibrosis extent, NT-proBNP levels and CMR-derived left ventricular global longitudinal strain impact on elevated pulmonary artery systolic pressure (PASP ⩾ 45 mm Hg) in patients with severe aortic stenosis.

Parameters/threshold	Area under Curve (95% CI)	Sensitivity (%)	Specificity (%)	SPAS <45/⩾45 mm Hg *n* (%)	p Value
MF > 10%	94.9 (88.8–101.8)	100	84.0	4 (16.0)/9 (100.0)	<0.001
LV GLS > −15.5%	86.3 (73.0–99.7)	100	82.6	19 (100)/0 (0)	<0.001
NT-proBNP > 2090 ng/l	85.1 (69–100.9)	85.7	88.9	3 (11.1)/6 (85.7)	<0.001

PASP: pulmonary artery systolic pressure; MF: extent of diffuse myocardial fibrosis; LV GLS: left ventricular global longitudinal strain; NT-pro BNP: N-terminal pro-hormone of brain natriuretic peptide.

## Discussion

The main finding of the present study is that histologically verified diffuse MF extent correlates with LV pathological remodeling—LV dilatation and dysfunction, which relates to PH in patients with severe AS.

AS is not only a disease of aortic valve, but also a disease of the ventricle. Long-standing pressure overload leads to LV hypertrophy and the presence of LV myocardial fibrosis plays a central part in the pathological LV remodeling.^17–20^ Treibel et al.^19^ demonstrated that both replacement and diffuse fibrosis correlates with LV dilatation, increased LV myocardial mass index and lower LV EF in patients with AS. Our study showed that not only LV dilation but also early LV dysfunction, mainly reduced GLS, is associated with diffuse myocardial fibrosis extent. In recent years, speckle tracking echocardiography (STE) and cardiac magnetic resonance mapping have been shown to provide complementary information for the assessment of left ventricular mechanics and identification of subtle damage by focal or diffuse MF, respectively.^7,8,21^ Our study confirmed that LV GLS parameters measured by FT-CMR method correlated with histologically verified MF (*r* = −0.5, p = 0.02). However, the data concerning relations of MF extent and PH in AS patients are scarce. Our study revealed that histologically proved diffused MF extent >10% is related to increased systolic pulmonary artery pressure with high sensitivity and specificity (respectively 100% and 84%, p < 0.01). However, histological verification of MF is not widely and not easily applied in everyday clinical practice, therefore the search of non-invasive indices of MF development is important. According to our data, LV GLS correlated not only with diffuse MF, but also with PH, and LV GLS > −15.5% was an independent predictor of PH in AS patients (p < 0.001). Our data coincides with Ahn et al.^22^ study, which concluded that reduced LV GLS, but not LV ejection fraction or radial and circumferential LV strain assessed by 2D-STE, related with PH in AS patients.

It should be noted that in our study no correlation was found between histologically verified diffuse and CMR detected LGE midventricular fibrosis (p = 0.08). LGE quantification is well validated in AS and supported by powerful adverse prognostic data, but is commonly considered as insensitive for the detection of diffuse interstitial fibrosis and acts as a marker of LV decompensation and irreversible LV changes.^30,31^ Combined, multiparametric approach with LGE and extracellular volume fraction quantification, as assessed by CMR T1 mapping, could improve early LV MF detection and quantification noninvasively, as well as its prognostic value on PH development and on clinical outcomes in AS patients.^9^

NT-proBNP is a widely used biomarker in clinical practice. Several studies demonstrated the role of this biomarker in noninvasive assessment of MF and the usefulness for prognosis of cardiovascular diseases such as heart failure, dilated and hypertrophic cardiomyopathy.^11,23^ In AS pressure overload induces MF that leads to increased chamber stiffness, diastolic dysfunction, and significant expression of NT-proBNP levels.^24–26^ Our study revealed the moderate correlation between larger diffuse MF extent and higher concentrations of this biomarker (*r* = 0.47, p = 0.005). As several studies concluded that MF plays a significant role in LV pathological remodeling and found the correlation of LV diameter, mass and dysfunction with NT-proBNP levels.^27–29^ This biomarker reflects the clinical and echocardiographic consequences of the left ventricular remodeling, as well as the elevated pulmonary artery systolic pressure.^28^ Multivariate analysis of our study demonstrated that NT-proBNP related to PH in patients with severe AS, and increased levels of this biomarker more than 2090 ng/ml was an independent predictor of PH with high sensitivity and specificity (respectively 85.7% and 88.9%, p < 0.01) in this patient group.

## Strengths and limitations

Combined histology and multimodality imaging study was performed in patients with AS, to find the association of MF not only with parameters of LV remodeling and dysfunction but also with PH, that plays important role for poor prognosis. The analysis of these studies was performed blinded by independent investigators groups.

The main limitation of this study was the small sample size, because the patients with other conditions related to PH (documented coronary artery disease or chronic obstructive pulmonary disease, atrial fibrillation, and moderate to severe mitral regurgitation) were excluded from the study. Secondly, pulmonary artery pressures were not measured by invasive cardiac catheterization.

## Conclusion

The extent of biopsy based diffuse MF together with reduced longitudinal left ventricular strain and increased levels of NT-proBNP relates to PH in patients with severe AS.

Further studies are needed to evaluate the combined, multiparametric approach with extracellular volume fraction quantification (ECV), LGE, and biopsy derived diffuse MF on LV pathological remodeling and the progression of PH in patients with severe aortic stenosis.
